# Distribution and Community Assembly of Trees Along an Andean Elevational Gradient

**DOI:** 10.3390/plants8090326

**Published:** 2019-09-05

**Authors:** Samantha J. Worthy, Rosa A. Jiménez Paz, Álvaro J. Pérez, Alex Reynolds, Jennifer Cruse-Sanders, Renato Valencia, John A. Barone, Kevin S. Burgess

**Affiliations:** 1 Department of Biology, Columbus State University, University System of Georgia, Columbus, GA 31907, USA (J.A.B.) (K.S.B.); 2 Department of Biology, University of Maryland, College Park, MD 20742, USA; 3 Laboratorio de Ecología de Plantas, Escuela de Ciencias Biológicas, Pontificia Universidad Católica del Ecuador, Quito 170143, Ecuador (R.A.J.P.) (R.V.); 4 Herbario QCA, Escuela de Ciencias Biológicas, Pontificia Universidad Católica del Ecuador, Quito 170143, Ecuador; 5 The Lovett School, Atlanta, GA 30327, USA; 6 State Botanical Garden of Georgia, Athens, GA 30605, USA

**Keywords:** community phylogenetics, Ecuador, montane forests, taxonomic metrics, tree diversity

## Abstract

Highlighting patterns of distribution and assembly of plants involves the use of community phylogenetic analyses and complementary traditional taxonomic metrics. However, these patterns are often unknown or in dispute, particularly along elevational gradients, with studies finding different patterns based on elevation. We investigated how patterns of tree diversity and structure change along an elevation gradient using taxonomic and phylogenetic diversity metrics. We sampled 595 individuals (36 families; 53 genera; 88 species) across 15 plots along an elevational gradient (2440–3330 m) in Ecuador. Seventy species were sequenced for the *rbcL* and *matK* gene regions to generate a phylogeny. Species richness, Shannon–Weaver diversity, Simpson’s Dominance, Simpson’s Evenness, phylogenetic diversity (PD), mean pairwise distance (MPD), and mean nearest taxon distance (MNTD) were evaluated for each plot. Values were correlated with elevation and standardized effect sizes (SES) of MPD and MNTD were generated, including and excluding tree fern species, for comparisons across elevation. Taxonomic and phylogenetic metrics found that species diversity decreases with elevation. We also found that overall the community has a non-random phylogenetic structure, dependent on the presence of tree ferns, with stronger phylogenetic clustering at high elevations. Combined, this evidence supports the ideas that tree ferns have converged with angiosperms to occupy the same habitat and that an increased filtering of clades has led to more closely related angiosperm species at higher elevations.

## 1. Introduction

Ecologists have long been interested in the distribution and assembly of plant communities. Broad trends and patterns have been described from local to global scales including well-known trends of decreasing species richness with increasing latitude and elevation [1,2,3]. Many of these trends and patterns have been documented in the temperate zone, but less so in the tropics, and even less in tropical montane forests due to their limited accessibility [4,5,6,7]. Recently, interest in investigating distribution and assembly patterns of plants in montane forests along elevational gradients has grown as these areas are home to at least a third of all terrestrial plant species and will likely show large effects from global warming [5,7,8,9,10].

Montane forests are an ideal system to study gradients in species composition because they eliminate confounding regional scale effects. These forests are also among the most species rich in the world, but consistently remain understudied compared to lowland tropical forests [7,8,11,12,13,14,15]. Montane forests are frequently immersed with clouds and are recognized for their low canopy height, multi-stemmed trees, and high epiphyte abundance [16,17,18]. They are also known to possess high levels of endemism, due in part to the unique environmental conditions and topography where they are found [7,12,15,19].

Traditionally, montane forest vegetation has been described using floristic inventories that calculate taxonomic diversity metrics such as species richness, Shannon–Weaver diversity [20], and Simpson’s Index [5,21,22,23,24]. These metrics have illuminated many globally consistent patterns of species distributions along elevation that have been useful in understanding the composition and diversity of plant species [25,26,27]. For example, alpha diversity has been shown to decrease with increasing elevation [19,28,29,30], but has also been found to have a hump-shaped pattern [6,11,31]. Interestingly, a number of abiotic and biotic factors such as temperature, precipitation, cloud cover, soil nutrients, light availability, and competition have been correlated with shifts in community composition along elevation [16,28,29,30,32]. Despite the success of these metrics, they alone struggle to describe montane forests’ biodiversity much less community assembly and species co-occurrence patterns along elevational gradients [10,33].

Community phylogenies have become increasingly important for providing additional information regarding the diversity and community assembly of forests beyond that which can be gained from analyzing species diversity and composition [33,34,35,36,37,38,39]. By merging understandings of ecology, evolution, and biogeography in plant communities, these phylogenies can reveal aspects of biodiversity that are not normally observable like linking phylogenetic diversity and dispersion to determine assembly mechanisms of forests [38,39,40,41,42,43]. Unlike taxonomic diversity metrics that use a nomenclatural approach, phylogenies allow an understanding of how communities evolved through time and offer further insights into historical and current processes contributing to diversity [10,33,44,45], hence, two communities could have the same species diversity, but different phylogenetic diversity [36]. To gain a better understanding of how montane forest communities are assembled, DNA barcodes can be used to construct phylogenies for community-level analyses. Phylogenies built using DNA barcodes are able to provide estimates of evolutionary distances and relationships between species within the phylogenies [24,42,46]. DNA barcodes used in the construction of plant phylogenies commonly include the phylogenetically conserved coding region, *rbcL*, combined with the more rapidly evolving gene region, *matK* [47,48,49]. Given the success of using DNA barcodes to build tropical forest community phylogenies [37,47,50,51], they can be established for less studied montane forests to highlight patterns of community assembly and structure not previously seen using taxonomic diversity metric values.

Community phylogenies can be used to investigate the assembly of plant biodiversity, which is thought to be due to a diverse array of abiotic and biotic mechanisms that filter species composition at both regional and local scales [52,53]. Community assembly patterns of phylogenetic relatedness typically fall into three categories: Random, clustered, or overdispersed based upon some attributes (e.g., relatedness, traits) of the species [34]. These categories focus on the rationale that some community assembly mechanisms favor co-occurrence of closely related species (phylogenetically clustered), whereas others favor co-occurrence of distantly related species (phylogenetically overdispersed) [35,42,49,51,54]. These categories can be used as a proxy to suggest underlying community assembly mechanisms [35,37,44]. Phylogenetic clustering has been hypothesized as evidence for the influence of habitat filtering (abiotic-driven processes) [37,46,47,54,55] or performance differences [56]. For phylogenetically overdispersed communities, biotic interactions (e.g., niche differences) are often hypothesized as important for local community assembly (e.g., competition) [33,37,45]. Often, however, the phylogenetic patterns underlying ecological and evolutionary mechanisms associated with composition of many plant communities remain unknown or in dispute [57]. For example, along elevation, some studies have found patterns of phylogenetic overdispersion [10,22,38,58], while others have found contrasting patterns of phylogenetic clustering [24,57]. Together, community phylogenetics and complementary species diversity metrics have the ability to detect important patterns of distribution, assembly, and structure of tree species along elevation in montane forests.

The goal of this study was to quantify patterns of tree species diversity and phylogenetic community assembly along an elevation gradient in montane forest and investigate potential ecological and evolutionary processes that underlie tree species co-occurrence. Specifically, we asked, 1) how do patterns of tree diversity and community structure change along an elevation gradient? 2) is there a relationship between diversity and structure trends found across elevation? and 3) how does the presence of tree ferns affect the phylogenetic community structure?

## 2. Results

### 2.1. Forest Composition

**Figure 1:**
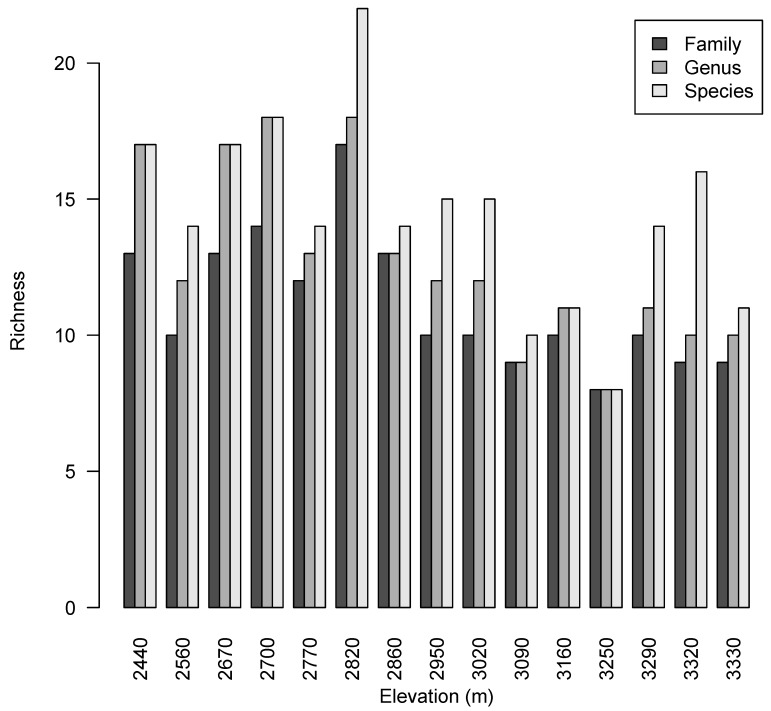
The richness of families, genera, and species within each plot in the transect established at the Siempre Verde Preserve, Imbabura Province, Ecuador.

**Figure 2:**
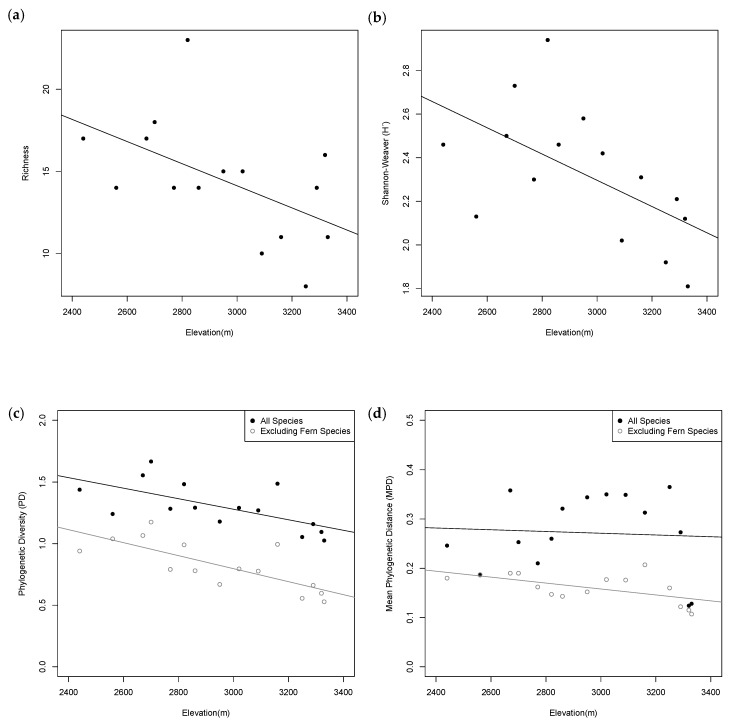

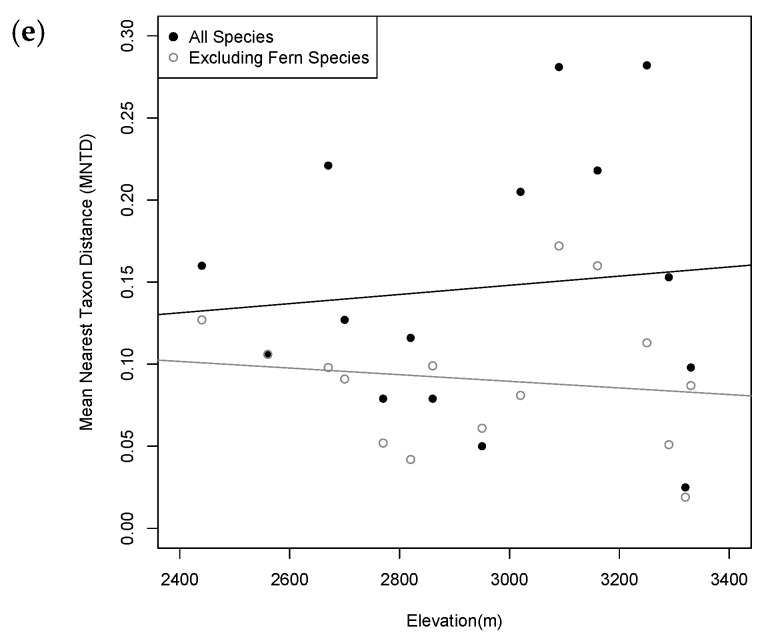
Relationship between diversity metrics and elevation. (**a**) Correlation between richness and elevation (r = −0.53, *p* = 0.04). (**b**) Correlation between Shannon–Weaver diversity (H´) and elevation (r = −0.58, *p* = 0.02). (**c**) Correlation between observed phylogenetic diversity (PD) and elevation with tree ferns (r = −0.65, *p* = 0.01) and without tree ferns (r = −0.76, *p* = 0.001). (**d**) Correlation between observed mean pairwise distance (MPD) and elevation with tree ferns (r = −0.06, *p* = 0.82) and without tree ferns (r = −0.58, *p* = 0.02). (**e**) Correlation between observed mean nearest taxon distance (MNTD) and elevation with tree ferns (r = 0.10, *p* = 0.72) and without tree ferns (r = −0.13, *p* = 0.64).

Within the transect, 595 individuals were tagged, collected, and identified. These comprised of 36 families, 53 genera, and 88 species (Figure 1, Table S1). Nine families make up 74.5% of the individuals of the entire transect, while 10 genera make up 67.2% (Figure 2, Table S1). By far, the two most abundant species in the transect are *Cyathea* cf. *frigida* (H. Karst.) Domin (*n* = 60) and *Weinmannia rollottii* Killip (*n* = 75). Plot number six at elevation 2820 m a.s.l. had the highest number of families (*n* = 17) and species (*n* = 22) of all the plots. It was also equal with plot four (2700 m a.s.l.) for the highest number of genera (*n* = 18).

### 2.2. Taxonomic Diversity Analysis

Richness, Shannon–Weaver diversity (H’), Simpson’s dominance (D_2_), and Simpson’s evenness (E) were calculated for each plot (Table 1). The highest H´ and D_2_ values were seen for plot six and the highest E was seen for plot 11 (Table 1). Pearson product-moment correlation tests were run between each diversity metric and elevation. Richness and H´ had significant negative correlations with elevation (Figure 2a,b). D_2_ nor E showed any significant correlation with elevation.

### 2.3. Phylogenetic Diversity Analysis

**Figure 3:**
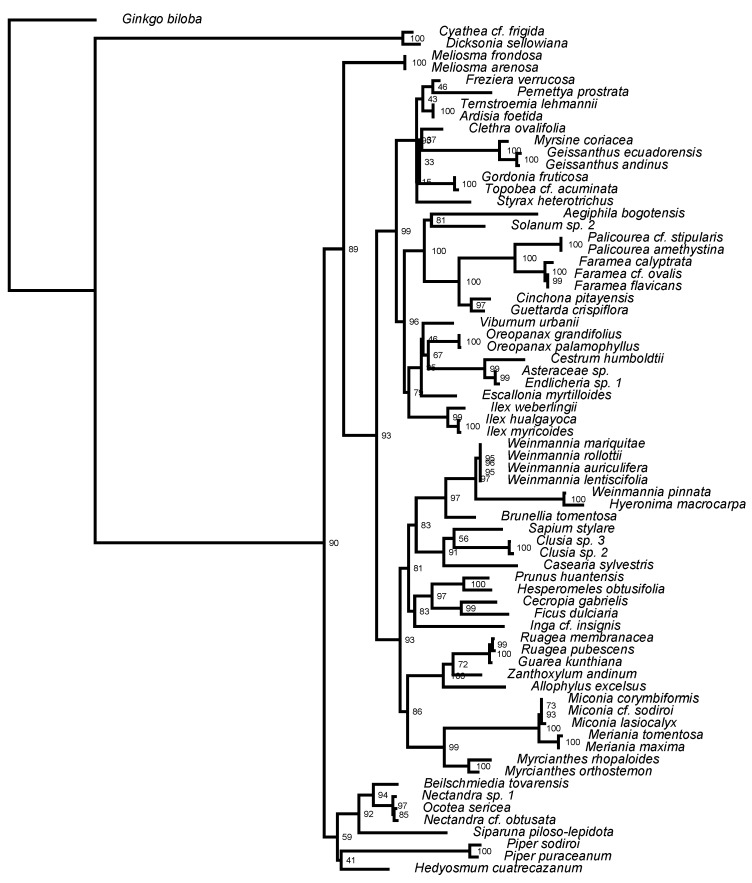
Phylogenetic tree of all species that were used in phylogenetic analyses from the transect at Siempre Verde Preserve, Imbabura Province, Ecuador. Bootstrap values based on maximum likelihood are reported at the nodes.

There were 35 families, 50 genera, and 70 species within the successfully sequenced data set (Table S2). DNA barcode sequence data was not recovered for 18 species (20.5%), but these species only accounted for 10.6% of the individuals in the transect. Of the 70 successfully sequenced individuals, 51 (72.9%) had both *rbcL* and *matK* sequences and 19 (27.1%) had only an *rbcL* sequence (Table S2). The consensus tree from rapid bootstrapping found 73.5% of all nodes were highly supported (bootstrap support >85%) and 85.3% of nodes showed moderate support (bootstrap support >70%; Figure 3). A significant negative correlation was found between observed values of phylogenetic diversity (PD) and elevation, but there was no correlation between the observed values mean pairwise distance (MPD) or mean nearest taxon distance (MNTD) and elevation (Figure 2c–e). Observed values were compared to null model calculations to determine significance. Across the three metrics, there were 17 instances of phylogenetic patterns significantly different from random, three cases of phylogenetic clustering (*p* < 0.05) and 14 cases of phylogenetic overdispersion (*p* > 0.95; Table 2; Tables S3–S5 for PD, MPD, and MNTD, respectively).

**Figure 4:**
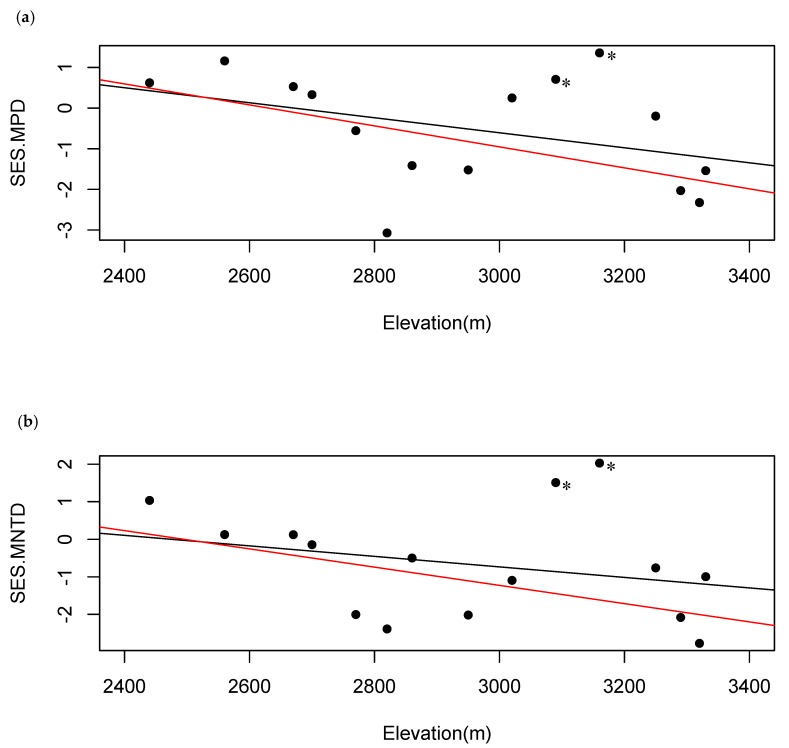
Relationship between standardized effect sizes (SES) of mean pairwise diversity (MPD) and mean nearest taxon distance (MNTD) and elevation with tree ferns excluded. (**a**) Black Line: Correlation between SES.MPD and elevation for all plots (r = −0.39, *p* = 0.15); Red Line: Correlation between SES.MPD and elevation when plots at 3090 m and 3160 m were excluded (r = −0.59, *p* = 0.03). (**b**) Black Line: Correlation between SES.MNTD and elevation for all plots (r = −0.28, *p* = 0.32); Red Line: Correlation between SES.MNTD and elevation when plots at 3090 m and 3160 m were excluded (r = −0.64, *p* = 0.02). Dots corresponding to the removed plots are noted with an *.

When tree ferns were excluded from the data set, phylogenetic patterns across the transect changed substantially. Across the three metrics, there were 13 instances of phylogenetic patterns significantly different from random, 11 cases of phylogenetic clustering (*p* < 0.05) and two cases of phylogenetic overdispersion (*p* > 0.95; Table 2; Tables S6–S8 for PD, MPD, and MNTD, respectively). A significant negative correlation was found between observed values of PD and elevation and observed values of MPD and elevation when tree ferns were excluded from the data (Figure 2c,d). No correlation was found between the observed values of MNTD and elevation when tree ferns were excluded (Figure 2e). For standardized effect sizes, SES.MPD and SES.MNTD, there was no correlation between values and elevation (Figure 4). However, a clear negative trend was visible and when off-trend plots 10 and 11 (3090 m and 3160 m) were removed, the correlations between SES.MPD and SES.MNTD and elevation became significant (Figure 4). Further investigation into the floras of these plots revealed low overlap of species between these plots and neighboring plots (Figure S1). Of the 17 species found in these two plots, 52% of them had a range along the elevation gradient that did not exceed these plots and 18% of these species were only found in these two plots.

## 3. Discussion

In this study, we investigated patterns of species distribution and structure across an elevational gradient using both taxonomic and phylogenetic metrics. Using multiple taxonomic metrics, we found evidence for species diversity decreasing with elevation (Figure 2a,b). We also found this pattern with observed phylogenetic diversity (PD), but not with observed mean pairwise distance (MPD; except when tree ferns were excluded) or observed mean nearest taxon distance (MNTD) metrics (Figure 2c–e). When considering the phylogenetic structure of the tree community, given the gradient of species diversity, we found a non-random structure that was contingent upon the presence of tree ferns. Without tree ferns, lower elevation communities exhibited similar phylogenetic structure as when tree ferns were included (Table 2; Tables S3–S8). However, at higher elevations, communities switched from patterns of phylogenetic overdispersion with tree ferns to phylogenetic clustering without tree ferns (Table 2; Tables S3–S8). Standardized effects sizes (SES) of MPD and MNTD were not significantly related with elevation, except when two off-trend plots were removed that showed evidence of colliding upper and lower elevation floras (Figure 4; Figure S1). Combined, this evidence supports the idea that tree ferns have converged with angiosperms to occupy the same habitat along with an increased filtering of clades at higher elevations.

### 3.1. Patterns of Taxonomic Distribution

This elevational transect comprised 595 individuals including 36 families, 53 genera, and 88 species (Figure 1, Table S1). The forest composition found at Siempre Verde is in general agreement with comparable studies conducted in other montane forest habitats. For example, a previous study found that South American montane forests are typically dominated by species of *Weinmannia, Schefflera, Miconia,* and *Myrcianthes* [59]. At the study site, we found that each of these genera, excluding *Schefflera*, were found to be among the most diverse within the transect (Table S1). Actually, *Weinmannia rollottii* (*n* = 75) is the most common species in the transect. In addition, the number of species (35) and families (19) in the high elevation plots were in-line with similar studies conducted along elevational gradients in the forests at Pasochoa volcano, Ecuador, where the number of species and families in high elevation plots were 32 and 21, respectively [60]. Furthermore, [11,28] found that Aquifoliaceae and Theaceae become more abundant at high elevations, while Melastomataceae is dominant at mid-elevations and Rubiaceae is common at lower elevations, results that match this study’s findings (Table S1).

Four separate analyses of community composition were performed: Richness, Shannon–Weaver diversity (H’), Simpson’s dominance (D_2_), and Simpson’s evenness. Only richness and H’ showed significant correlations with elevation (Figure 2a,b, Table 1). Both richness and H’ decreased as elevation increased. This tendency of decreasing diversity has been shown along elevational gradients in different forest types around the world [7,28,29,30,61,62,63], although, regardless of the trend, we found the highest values for both metrics at mid-elevation (plot six, 2820 m a.s.l.). Other studies along elevational gradients have found similar findings where a plot, not located at the lowest elevation, exceeds all others in diversity [6,11,19,31]. Cloud cover may be one potential cause for this mid-elevation increase in diversity. Cloud cover is known to saturate montane forests causing a decrease in temperature and an increase in precipitation and overall moisture [6,16,18,32]. This has led many to refer to plots located where clouds move into the forest as “mid-elevation bulges” as the highest diversity is often seen at these intermediate elevation sites [6,7,64]. It has been hypothesized that at these mid-elevations, a mixture of species from low and high elevations have reached the maximum and minimum, respectively, of their elevational range and have converged on a particular niche that combines the effects of the environment and competition, increasing diversity [9,11,24,31].

### 3.2. Patterns of Phylogenetic Distribution

Sequence recovery rates at our study site were relatively high, where we obtained a genetic sequence for ~80% of species located within the elevational transect, and with 72.9% of those having both *rbcL* and *matK* sequences and 27.1% missing the *matK* sequence (Table S2). This recovery rate is slightly lower compared to similar studies, where in tropical and temperate forests, other studies have successfully sequenced between 85–93% of samples for *rbcL* and between 69–75% of samples for *matK* [47,50,51,65]. The higher recovery rate for *rbcL* over that of *matK* has been shown to be attributable to its shorter length and better capability of sequencing across all angiosperms making it easier to obtain [37]. In our study, DNA samples were taken from herbarium specimens at Herbario QCA at Pontificia Universidad Católica del Ecuador that had been preserved in alcohol. It is known that alcohol quickly degrades the quality of DNA [66,67], which also may have led to the slight reduction in sequence recovery seen here compared to other studies. DNA vouchers should be taken from fresh collections and dried in silica gel until DNA extraction. Currently there is a lack of publicly available barcode sequences for montane plant species. Our collection represents a substantial contribution to public reference databases, as the majority of the species in our study were not accessible for research.

Comparisons between patterns of phylogenetic community structure with and without tree fern species revealed the impact the abundance of these tree ferns had on driving the phylogenetic patterns. Observed values of each of the phylogenetic diversity metrics were tested for correlation with elevation as a proxy for environmental variables known to change with elevation. With and without tree ferns present, PD was significantly negatively correlated with elevation (Figure 2c), which is expected as this metric is the sum of all the branch lengths in the phylogeny and thus as species richness decreases as elevation increases there are fewer branches in the phylogeny [38,68,69]. However, when tree ferns were excluded, observed values of MPD were also negatively correlated with elevation (Figure 2d). This suggests that if we exclude tree ferns, communities at higher elevations are made up of less diverse, more closely related species, a commonly found pattern [24,70,71].

With tree ferns included, standardized effect sizes of phylogenetic distance (PD), mean pairwise distance (MPD), and mean nearest taxon distance (MNTD), revealed phylogenetic overdispersion in 14 instances, phylogenetic clustering in three instances, and phylogenetic randomness in all other cases indicating a lack of uniform phylogenetic structure across the elevation gradient (Table 2). However, without tree ferns, these patterns were drastically altered with 11 instances of phylogenetic clustering and two instances of phylogenetic overdispersion (Table 2). For example, plot 6 (2820 m a.s.l.) showed no significant phylogenetic pattern when tree ferns were included in the analyses, but showed significant phylogenetic clustering across all three metrics (SES.PD, SES.MPD, and SES.MNTD) when tree ferns were excluded (Table 2). Few plots remained consistent in their phylogenetic pattern with and without tree ferns. Plot 14 (3320 m a.s.l.) did remain consistent and showed significant clustering for SES.MPD and SES.MNTD with and without tree ferns in the analyses. This suggests that co-occurring species are more closely related than expected by chance. This result is not surprising given that 53 of the 100 stems in this plot are from the genus *Weinmannia* within the family Cunoniaceae. In general, there is a non-random phylogenetic structure along the elevation gradient, with discrepancies between low and high elevation plots that are largely influenced by the presence or absence of tree fern species.

Both with and without tree ferns, standardized effects sizes (SES) of MPD and MNTD were not significantly related to elevation. However, without tree ferns, a visible negative trend for both metrics with elevation was obvious (Figure 4). Our results agreed with prior research findings of increased phylogenetic clustering at higher elevations; hypothesized to be evidence for the influence of abiotic driven processes on phylogenetic community structure [24,57,72,73]. The relationships between SES.MPD, SES.MNTD, and elevation were significant when two off-trend plots were removed (Figure 4; Figure S1). We found that the floras of these two plots were distinct from neighboring floras with multiple species found here exclusively or not exceeding this elevation along the transect. This could be evidence of colliding floras from upper and lower elevations, but more extensive sampling is needed for further investigation. In total, our results support ideas of habitat convergence by tree ferns with angiosperms and an increased filtering of clades leading to phylogenetic clustering at higher elevations.

## 4. Materials and Methods

### 4.1. Study Site

This study was conducted at the Siempre Verde Preserve in the Imbabura Province of northern Ecuador between 2014 and 2016. Siempre Verde is located in the western foothills of the Cotacachi volcano in Andean forest in the eastern most portion of the Intag River Valley (00°22’38”N, 78°25’37”W). The preserve covers 504 hectares and has an elevation range from 2350 to 3330 m above sea level [74]. At Siempre Verde, the rainy season begins in October and ends in June. The area receives ~2532 mm of annual rainfall with the heaviest rains happening between January and April. The driest months are usually between July and September [74]. The large temperature range at the preserve is due to the steep elevational cline. At intermediate elevations (~2460 m), the temperature ranges from ~6.4 °C to 24.2 °C. At the top of the mountain, the range is ~4.5 °C to 18 °C [74]. According to the General Soil Map of Ecuador [75], the soil at Siempre Verde is allophanic, loam to silty loam and deeply rich in organic material. The soil is of medium fertility and has an acidic pH with low base saturation (20–100%) [75]. These soils are the result of slow weathering of volcanic ash and glass, especially at high elevations in the tropical Andes [74].

### 4.2. Sampling Design

A transect was established, which included 15 plots that were 5 m × 50 m (0.025 ha each). The plots were at approximately 100 m intervals in distance, from 2440 to 3330 m a.s.l. Within each plot, every tree and tree fern with a diameter at breast height (dbh = 130 cm) of ≥ 5 cm was tagged with a numbered aluminum plate (Table S1) [76]. Samples were collected from each individual for identification and layered in newspaper in a plant press and soaked with alcohol to control for pests until placed in a plant dryer, common practice in tropical plant collecting. Herbarium specimens were deposited into the Herbario QCA at Pontificia Universidad Católica del Ecuador [76,77]. Plots were not replicated in an additional transect in order to minimize the effect of aspect due to the complex topography of the site (see [7,19,63] for similar methods).

### 4.3. DNA Isolation, PCR Amplification, and Sequencing

DNA extraction [78,79], PCR amplification [80,81], and sequencing [82,83] were performed with semiautomated protocols at the Canadian Centre for DNA Barcoding, Biodiversity Institute of Ontario, Canada. Dried plant tissue (1–5 mg) was ground using a TissueLyser II (QIAGEN, Germantown, Maryland, USA) at 28 Hz for 60–90 seconds at room temperature using the Axygen Mini Tube System (Axygen Scientific, Union City, California, USA) with one 3.17 mm stainless steel bead per tube. Cells were then lysed with 250–400 µL of 2× cetyltrimethylammonium bromide (CTAB) buffer and incubated at 65 °C for 60–90 minutes. Then, 50 µL of lysate was transferred into a 96-well microplate (Eppendorf, Hamburg, Germany) using a Liquidator 96 (Mettler Toledo, Mississauga, Ontario, Canada). Isolation and purification of DNA was done by binding to glass fiber filtration columns [78] on a Biomek FX Workstation (Beckman Coulter, Mississauga, Ontario, Canada). DNA concentrations of 20–40 ng/µL were generated and used for PCR amplification with Platinum DNA Polymerase (Invitrogen, Carlsbad, California, USA; for protocols see: [80,81]). Two coding gene regions of the chloroplast genome were sequenced using forward and reverse primers: The phylogenetically conserved ribulose-bisphosphate/carboxylase large subunit (*rbcL)* gene region [47,84] and the more rapidly evolving region, maturase-K (*matK)* gene region [85,86] (Table S9). Using Sanger sequencing technology, the sequencing products were read via laser electrophoresis in a 3730 xl DNA Analyzer [82]. Due to low sequence recovery, specimens that did not generate a sequence were resampled and the processes of DNA isolation, PCR amplification, and sequencing were repeated for each sample.

### 4.4. Data Analysis

#### 4.4.1. Community Composition

To quantify alpha diversity trends along the transect, three taxonomic diversity metrics were calculated: Shannon–Weaver [20], Simpson’s Dominance [21], and Simpson’s Evenness. Shannon–Weaver diversity (H’) estimates the average uncertainty of the identity of an unknown individual [87,88]. This metric stresses richness and responds strongest to changes in importance of the rarest species in the community [87,88]. Simpson’s dominance (D_2_) is the inverse of Simpson’s diversity representing the probability that two randomly selected individuals belong to different species [88]. This metric responds most strongly to changes in proportional abundance of the most common species [87]. Lastly, Simpson’s evenness (E) represents the relative abundance of species in an area with higher values indicating more even distribution of individuals among species and thus higher diversity of the area [88]. It was calculated as Simpson’s dominance divided by richness [88]. Shannon–Weaver and Simpson’s dominance indices were calculated using the vegan package [89] in the R programming language [90]. Pearson product-moment correlation tests were run between each diversity metric and elevation with significance determined at *p* < 0.05 in all cases.

#### 4.4.2. Phylogenetic Analysis

After sequences for each species were obtained, alignments and phylogenies were constructed using Geneious version 10.2.6 (http://www.geneious.com) [91]. The *rbcL* and *matK* genes were aligned separately using multiple alignment and fast Fourier transform (MAFFT v. 7.388) implemented in Geneious version 10.2.6 [92,93] with the FFT-NS-2 option with each alignment then concatenated into a supermatrix. A phylogeny was generated applying maximum likelihood (ML) methods, using randomized axelerated maximum likelihood (RAxML v. 8.2.11) [94,95]. *Ginkgo biloba* served as the outgroup, in accordance with other research [47,96,97], and nucleotide substitution was modeled using the general time reversible model with gamma-distributed rate variation across sites (GTR + GAMMA model), with substitution rates estimated independently for each gene [49,51]. The rapid bootstrapping algorithm was implemented to search for the best scoring ML tree after node support was evaluated using 1000 bootstrap runs [98].

#### 4.4.3. Phylogenetic Structure Analysis

All phylogenetic analyses were estimated within the Picante package [99] of the R programming language [90]. Three metrics were assessed in this study, phylogenetic diversity (PD) [100], mean pairwise distance (MPD) [34], and mean nearest taxon distance (MNTD) [34]. Faith’s PD is the sum of all branch lengths in an assemblage (1):

(1) $${\mathrm{Faith}}^{\prime }s\;\mathrm{PD}\;=\sum_{i}^{n}l_{i},$$

where n is the number of branches in the phylogenetic tree and the length of the ith branch is $l_{i}$ [68,69]. PD values can be correlated with species richness in a system because adding a species would also add, at a minimum, a terminal branch to the phylogeny, thus altering the PD value [46].

The MPD metric obtains a pairwise phylogenetic distance across all pairs of taxa in a community and gives an estimate of the overall divergence of taxonomic clades present using Equation (2):

(2) $$\mathrm{Abundance}\;\mathrm{Weighted}\;\mathrm{MPD}\;=\frac{\sum_{i}^{n}\sum_{j}^{n}\delta_{i,j}f_{i}f_{j}}{\sum_{i}^{n}\sum_{j}^{n}f_{i}f_{j}},\mathrm{where}\;i\neq j,$$

where there are n species in the community, $\delta_{i,j}$ is the phylogenetic distance between species i and j, and f represents the frequency of species. It can be considered a “basal” metric of phylogenetic diversity as it captures the overall phylogenetic dissimilarity of the taxa in a sample. MPD does not detect finer scale phylogenetic patterns that may be present [46,68].

The last metric estimated was MNTD. It provides an average of the distance between each species and its nearest phylogenetic neighbor in the community. It quantifies the degree that a community may be a set of closely related species versus a heterogeneous set of taxa from disparate taxonomic clades using Equation (3) [46]:

(3) $$\mathrm{Abundance}\;\mathrm{Weighted}\;\mathrm{MNTD}\;=\frac{\sum_{i}^{n}{\min\delta }_{i,j}f_{i}}{n},\;\mathrm{where}\;i\neq j,$$

where there are n species in the community, $\delta_{i,j}$ is the phylogenetic distance between species i and j, and min $\delta_{i,j}$ is the minimum phylogenetic distance between species i and all other species in the community (i.e., the nearest neighbor distance). The variable $f_{i}\;$(frequency) was included to indicate the abundance of species i in the community [68].

As the raw values of PD, MPD, and MNTD give no means of standardized comparisons between communities (i.e., whether these measures are different from the expected given the observed species richness), null models were implemented so that standardized effect sizes (SES) could be determined using Equation (4):

(4) $$\mathrm{Standardized}\;\mathrm{Effect}\;\mathrm{Sizes}\;=\frac{\mathrm{observed}-\;\bar{\mathrm{null}}}{\mathrm{sd}(\mathrm{null})}.$$

This calculation removes any directional bias associated with the decreases in variance in the expected values with increasing species richness [68]. For MPD and MNTD, positive SES values (obs.z > 0) and high quantiles (obs.p > 0.95) indicate phylogenetic overdispersion, or a greater phylogenetic distance among co-occurring species than expected. Negative SES values (obs.z < 0) and low quantiles (obs.p < 0.05) indicate phylogenetic clustering, or smaller phylogenetic distances among co-occurring species than expected [49,51,101]. To conduct the null modeling, we randomized the names of species across the tips of the phylogeny and re-calculated each metric. This was repeated 999 times. This null model only randomizes relatedness and fixes all observed patterns in the community data matrix (e.g., species richness, occupancy rates, and abundance distributions).

Two tree fern species are present in the transect, *Dicksonia sellowiana* and *Cyathea* cf. *frigida*, the second most abundant species in the transect. They are distantly related to the other species in the transect and could have a disproportionately large impact on patterns of phylogenetic structure along the transect. To address this, SES.PD, SES.MPD, and SES.MNTD were recalculated after dropping these two tree fern species from the phylogeny. Pearson product-moment correlation tests were run between observed and standardized effect size values of PD, MPD, and MNTD and elevation, a surrogate for abiotic factors that co-vary with elevation, with significance determined at *p* < 0.05 in all cases.

## 5. Conclusions

Accessing diversity and community assembly of montane forests is critically important as they are increasingly under anthropogenic pressures [8,12]. These forests are havens for endemic species and those that are shifting their ranges for climate adaptation, in particular, should be a conservation priority [7,12,102]. As most of the research on montane forests has focused in the Neotropics, further effort should be put into expanding research efforts to other study regions to better characterize the composition and structure of montane forests along elevational gradients on a global scale [8,18]. To aid the comprehensive understanding of montane forests, broader relationships that consider climate, geology, soils, and vegetation types, require further study.

## Figures and Tables

**Table 1:** Shannon–Weaver Diversity (H’), Simpson’s Dominance (D_2_), and Simpson’s Evenness (E) for each plot (1–15) of the transect. The elevation of each plot, as well as the total number of stems, species, genera, and families, are given.

Plot	Elevation (m)	Number of Stems	Species Richness	Genus Richness	Family Richness	Species H´	Species D_2_	Species E
1	2440	53	17	17	13	2.46	8.59	0.51
2	2560	42	14	12	10	2.13	5.62	0.40
3	2670	33	17	17	13	2.50	8.57	0.50
4	2700	29	18	18	14	2.73	12.94	0.72
5	2770	39	14	13	12	2.30	7.80	0.56
6	2820	44	23	18	17	2.94	15.87	0.69
7	2860	23	14	13	13	2.46	9.62	0.69
8	2950	24	15	12	10	2.58	11.52	0.77
9	3020	35	15	12	10	2.42	8.81	0.59
10	3090	21	10	9	9	2.02	5.88	0.59
11	3160	21	11	11	10	2.31	9.38	0.85
12	3250	17	8	8	8	1.92	5.90	0.74
13	3290	63	14	11	10	2.21	6.98	0.50
14	3320	100	16	10	9	2.12	5.23	0.33
15	3330	51	11	10	9	1.81	4.40	0.40

**Table 2:** Observed values for three phylogenetic diversity metrics, phylogenetic distance (PD), mean pairwise distance (MPD), and mean nearest taxon distance (MNTD), with and without tree ferns, are given for each plot. For each metric, 999 randomizations were used to assess departure from random. Significant differences from random are in bold. The * denotes a significant overdispersion pattern *(p* > 0.95) and the ^ denotes a significant clustering pattern *(p* < 0.05).

Plot	Elevation (m)	PD	PD: No Ferns	MPD	MPD: No Ferns	MNTD	MNTD: No Ferns
1	2440	1.438	0.940	0.246	0.180	0.160	0.127
2	2560	1.241	1.039	0.186	0.187	0.106	0.106
3	2670	1.555	1.066	0.358 *	0.190	0.221 *	0.098
4	2700	1.667 *	1.175	0.253	0.190	0.127	0.091
5	2770	1.283	0.791	0.210	0.162	0.079	0.052 ^
6	2820	1.483	0.990^	0.260	0.147 ^	0.116	0.042 ^
7	2860	1.292	0.780	0.321 *	0.143	0.079	0.099
8	2950	1.179	0.669	0.344 *	0.152	0.050 ^	0.061 ^
9	3020	1.290	0.795	0.350 *	0.177	0.205 *	0.081
10	3090	1.270	0.777	0.349 *	0.176	0.281 *	0.172
11	3160	1.487 *	0.994 *	0.313 *	0.207	0.218 *	0.160 *
12	3250	1.054	0.556	0.365 *	0.160	0.282 *	0.113
13	3290	1.159	0.662	0.273	0.122^	0.153	0.051 ^
14	3320	1.095	0.597 ^	0.124 ^	0.115^	0.025 ^	0.019 ^
15	3330	1.025	0.528 ^	0.128	0.107	0.098	0.087

## Supplementary Materials

The following are available online at https://www.mdpi.com/2223-7747/8/9/326/s1, Figure S1: Number of overlapping species between pairs of plots along the elevational gradient, Table S1: All 595 individuals found within the transect at Siempre Verde Preserve, Imbabura Province, Ecuador, that were tagged, collected and identified, Table S2: Specimen information for samples included in the phylogenetic analyses, Table S3: Standard effects sizes for phylogenetic diversity (PD) randomizations for each plot, Table S4: Standard effects sizes for mean pairwise distance (MPD) randomizations for each plot, Table S5: Standard effects sizes for mean nearest taxon distance (MNTD) randomizations for each plot, Table S6: Standard effects sizes for phylogenetic diversity (PD) randomizations for each plot excluding tree ferns from the data, Table S7: Standard effects sizes for mean pairwise distance (MPD) randomizations for each plot excluding tree ferns from the data, Table S8: Standard effects sizes for mean nearest taxon distance (MNTD) randomizations for each plot excluding tree ferns from the data, Table S9: Forward and reverse primer sequences for the *rbcL* and *matK* gene regions used for sequencing in this study.
