# Supplementary material for: Distribution and Community Assembly of Trees Along an Andean Elevational Gradient
Source: Plants (Basel). 2019 Sep 5;8(9):326. doi: 10.3390/plants8090326 (PMC6783956; doi:10.3390/plants8090326)
Supplement: Supplementary file 1 [file plants-08-00326-s001.pdf]

plants

MDPI

Table 1. All 595 individuals found within the transect at Siempre Verde Preserve, Imbabura Province, Ecuador, were tagged, collected and identified. They are organized alphabetically by family, then genus, then species names. The elevation (m) of the plot in which the individual is found in and that individual's plant number are included.

Family	Genus	Specific Epithet	Altitude	Plant Number
Adoxaceae	*Viburnum*	*urbanii*	3250	375
Adoxaceae	*Viburnum*	*urbanii*	3160	357
Adoxaceae	*Viburnum*	*urbanii*	3160	361
Adoxaceae	*Viburnum*	*urbanii*	2820	226
Aquifoliaceae	*Ilex*	*hualgayoca*	3320	499
Aquifoliaceae	*Ilex*	*hualgayoca*	3320	534
Aquifoliaceae	*Ilex*	*hualgayoca*	3320	553
Aquifoliaceae	*Ilex*	*hualgayoca*	3320	567
Aquifoliaceae	*Ilex*	*hualgayoca*	3320	585
Aquifoliaceae	*Ilex*	*hualgayoca*	3320	588
Aquifoliaceae	*Ilex*	*hualgayoca*	3330	463
Aquifoliaceae	*Ilex*	*hualgayoca*	3330	485
Aquifoliaceae	*Ilex*	*hualgayoca*	3330	486
Aquifoliaceae	*Ilex*	*hualgayoca*	3330	487
Aquifoliaceae	*Ilex*	*hualgayoca*	3330	488
Aquifoliaceae	*Ilex*	*hualgayoca*	3330	489
Aquifoliaceae	*Ilex*	*hualgayoca*	3330	490
Aquifoliaceae	*Ilex*	*hualgayoca*	3330	491
Aquifoliaceae	*Ilex*	*hualgayoca*	3330	492
Aquifoliaceae	*Ilex*	*hualgayoca*	3330	493
Aquifoliaceae	*Ilex*	*hualgayoca*	3330	494
Aquifoliaceae	*Ilex*	*hualgayoca*	3330	495
Aquifoliaceae	*Ilex*	*hualgayoca*	3290	402
Aquifoliaceae	*Ilex*	*hualgayoca*	3290	439
Aquifoliaceae	*Ilex*	*hualgayoca*	2820	210
Aquifoliaceae	*Ilex*	*myricoides*	3320	503
Aquifoliaceae	*Ilex*	*myricoides*	3320	517
Aquifoliaceae	*Ilex*	*myricoides*	3330	474
Aquifoliaceae	*Ilex*	*weberlingii*	3320	587
Araliaceae	*Oreopanax*	*grandifolius*	3160	362
Araliaceae	*Oreopanax*	*grandifolius*	2860	253
Araliaceae	*Oreopanax*	*palamophyllus*	3090	326
Araliaceae	*Oreopanax*	*palamophyllus*	3090	330
Araliaceae	*Oreopanax*	*palamophyllus*	2700	139
Araliaceae	*Oreopanax*	*palamophyllus*	2700	140
Araliaceae	*Oreopanax*	*palamophyllus*	2700	145
Araliaceae	*Oreopanax*	*palamophyllus*	2700	148
Araliaceae	*Oreopanax*	*palamophyllus*	2670	120
Asteraceae	*Asteraceae*	*sp*	2860	242
Asteraceae	*Asteraceae*	*sp*	2820	202
Asteraceae	*Asteraceae*	*sp*	2440	5
Asteraceae	*Asteraceae*	*sp*	2440	40
Brunelliaceae	*Brunellia*	*acostae*	2700	154
Brunelliaceae	*Brunellia*	*tomentosa*	3250	374

Chloranthaceae	*Hedyosmum*	*cuatrecazanum*	2820	216
Chloranthaceae	*Hedyosmum*	*cuatrecazanum*	2770	159
Chloranthaceae	*Hedyosmum*	*cuatrecazanum*	2770	161
Chloranthaceae	*Hedyosmum*	*cuatrecazanum*	2770	172
Chloranthaceae	*Hedyosmum*	*cuatrecazanum*	2770	177
Chloranthaceae	*Hedyosmum*	*cuatrecazanum*	2770	194
Chloranthaceae	*Hedyosmum*	*cuatrecazanum*	2700	152
Chloranthaceae	*Hedyosmum*	*cuatrecazanum*	2670	96
Chloranthaceae	*Hedyosmum*	*cuatrecazanum*	2670	109
Clethraceae	*Clethra*	*ovalifolia*	3320	502
Clethraceae	*Clethra*	*ovalifolia*	3320	529
Clethraceae	*Clethra*	*ovalifolia*	3320	530
Clethraceae	*Clethra*	*ovalifolia*	3320	538
Clethraceae	*Clethra*	*ovalifolia*	3320	589
Clethraceae	*Clethra*	*ovalifolia*	3320	596
Clusiaceae	*Clusia*	*sp1*	3020	299
Clusiaceae	*Clusia*	*sp1*	2950	273
Clusiaceae	*Clusia*	*sp1*	2860	245
Clusiaceae	*Clusia*	*sp1*	2820	228
Clusiaceae	*Clusia*	*sp1*	2820	235
Clusiaceae	*Clusia*	*sp1*	2820	240
Clusiaceae	*Clusia*	*sp2*	2560	70
Clusiaceae	*Clusia*	*sp2*	2560	71
Clusiaceae	*Clusia*	*sp3*	2860	255
Clusiaceae	*Clusia*	*sp3*	2820	221
Clusiaceae	*Clusia*	*sp5*	2670	119
Clusiaceae	*Clusia*	*sp5*	2670	123
Clusiaceae	*Clusia*	*sp5*	2560	58
Clusiaceae	*Clusia*	*sp5*	2560	66
Clusiaceae	*Clusia*	*sp5*	2560	79
Clusiaceae	*Clusia*	*sp5*	2440	2
Clusiaceae	*Clusia*	*sp5*	2440	21
Clusiaceae	*Clusia*	*sp5*	2440	24
Clusiaceae	*Clusia*	*sp5*	2440	30
Cunoniaceae	*Weinmannia*	*auriculifera*	3320	500
Cunoniaceae	*Weinmannia*	*auriculifera*	3320	509
Cunoniaceae	*Weinmannia*	*auriculifera*	3320	513
Cunoniaceae	*Weinmannia*	*auriculifera*	3320	524
Cunoniaceae	*Weinmannia*	*auriculifera*	3320	525
Cunoniaceae	*Weinmannia*	*auriculifera*	3320	537
Cunoniaceae	*Weinmannia*	*auriculifera*	3320	546
Cunoniaceae	*Weinmannia*	*auriculifera*	3290	420
Cunoniaceae	*Weinmannia*	*auriculifera*	3290	428
Cunoniaceae	*Weinmannia*	*auriculifera*	3290	433
Cunoniaceae	*Weinmannia*	*auriculifera*	2950	284
Cunoniaceae	*Weinmannia*	*lentiscifolia*	3250	367
Cunoniaceae	*Weinmannia*	*lentiscifolia*	3250	370
Cunoniaceae	*Weinmannia*	*lentiscifolia*	3250	376
Cunoniaceae	*Weinmannia*	*lentiscifolia*	3090	331
Cunoniaceae	*Weinmannia*	*lentiscifolia*	3020	288
Cunoniaceae	*Weinmannia*	*lentiscifolia*	3020	289
Cunoniaceae	*Weinmannia*	*lentiscifolia*	3020	294

Cunoniaceae	*Weinmannia*	*lentiscifolia*	3020	303
Cunoniaceae	*Weinmannia*	*lentiscifolia*	3020	311
Cunoniaceae	*Weinmannia*	*lentiscifolia*	3020	322
Cunoniaceae	*Weinmannia*	*lentiscifolia*	2950	264
Cunoniaceae	*Weinmannia*	*lentiscifolia*	2950	279
Cunoniaceae	*Weinmannia*	*lentiscifolia*	2950	285
Cunoniaceae	*Weinmannia*	*lentiscifolia*	2820	198
Cunoniaceae	*Weinmannia*	*lentiscifolia*	2770	163
Cunoniaceae	*Weinmannia*	*mariquitae*	3320	531
Cunoniaceae	*Weinmannia*	*mariquitae*	3320	573
Cunoniaceae	*Weinmannia*	*mariquitae*	3320	595
Cunoniaceae	*Weinmannia*	*pinnata*	3320	527
Cunoniaceae	*Weinmannia*	*pinnata*	3320	541
Cunoniaceae	*Weinmannia*	*pinnata*	3320	561
Cunoniaceae	*Weinmannia*	*pinnata*	3320	562
Cunoniaceae	*Weinmannia*	*pinnata*	3320	563
Cunoniaceae	*Weinmannia*	*pinnata*	3160	347
Cunoniaceae	*Weinmannia*	*pinnata*	3160	356
Cunoniaceae	*Weinmannia*	*pinnata*	3160	359
Cunoniaceae	*Weinmannia*	*rollottii*	3320	501
Cunoniaceae	*Weinmannia*	*rollottii*	3320	504
Cunoniaceae	*Weinmannia*	*rollottii*	3320	505
Cunoniaceae	*Weinmannia*	*rollottii*	3320	512
Cunoniaceae	*Weinmannia*	*rollottii*	3320	516
Cunoniaceae	*Weinmannia*	*rollottii*	3320	518
Cunoniaceae	*Weinmannia*	*rollottii*	3320	522
Cunoniaceae	*Weinmannia*	*rollottii*	3320	528
Cunoniaceae	*Weinmannia*	*rollottii*	3320	533
Cunoniaceae	*Weinmannia*	*rollottii*	3320	536
Cunoniaceae	*Weinmannia*	*rollottii*	3320	540
Cunoniaceae	*Weinmannia*	*rollottii*	3320	542
Cunoniaceae	*Weinmannia*	*rollottii*	3320	543
Cunoniaceae	*Weinmannia*	*rollottii*	3320	544
Cunoniaceae	*Weinmannia*	*rollottii*	3320	547
Cunoniaceae	*Weinmannia*	*rollottii*	3320	548
Cunoniaceae	*Weinmannia*	*rollottii*	3320	549
Cunoniaceae	*Weinmannia*	*rollottii*	3320	551
Cunoniaceae	*Weinmannia*	*rollottii*	3320	552
Cunoniaceae	*Weinmannia*	*rollottii*	3320	554
Cunoniaceae	*Weinmannia*	*rollottii*	3320	555
Cunoniaceae	*Weinmannia*	*rollottii*	3320	556
Cunoniaceae	*Weinmannia*	*rollottii*	3320	559
Cunoniaceae	*Weinmannia*	*rollottii*	3320	568
Cunoniaceae	*Weinmannia*	*rollottii*	3320	569
Cunoniaceae	*Weinmannia*	*rollottii*	3320	572
Cunoniaceae	*Weinmannia*	*rollottii*	3320	574
Cunoniaceae	*Weinmannia*	*rollottii*	3320	576
Cunoniaceae	*Weinmannia*	*rollottii*	3320	577
Cunoniaceae	*Weinmannia*	*rollottii*	3320	578
Cunoniaceae	*Weinmannia*	*rollottii*	3320	580
Cunoniaceae	*Weinmannia*	*rollottii*	3320	581
Cunoniaceae	*Weinmannia*	*rollottii*	3320	582

Cunoniaceae	*Weinmannia*	*rollottii*	3320	586
Cunoniaceae	*Weinmannia*	*rollottii*	3320	590
Cunoniaceae	*Weinmannia*	*rollottii*	3320	591
Cunoniaceae	*Weinmannia*	*rollottii*	3320	592
Cunoniaceae	*Weinmannia*	*rollottii*	3320	594
Cunoniaceae	*Weinmannia*	*rollottii*	3330	447
Cunoniaceae	*Weinmannia*	*rollottii*	3330	449
Cunoniaceae	*Weinmannia*	*rollottii*	3330	453
Cunoniaceae	*Weinmannia*	*rollottii*	3330	454
Cunoniaceae	*Weinmannia*	*rollottii*	3330	457
Cunoniaceae	*Weinmannia*	*rollottii*	3330	459
Cunoniaceae	*Weinmannia*	*rollottii*	3330	461
Cunoniaceae	*Weinmannia*	*rollottii*	3330	462
Cunoniaceae	*Weinmannia*	*rollottii*	3330	464
Cunoniaceae	*Weinmannia*	*rollottii*	3330	468
Cunoniaceae	*Weinmannia*	*rollottii*	3330	469
Cunoniaceae	*Weinmannia*	*rollottii*	3330	470
Cunoniaceae	*Weinmannia*	*rollottii*	3330	471
Cunoniaceae	*Weinmannia*	*rollottii*	3330	472
Cunoniaceae	*Weinmannia*	*rollottii*	3330	473
Cunoniaceae	*Weinmannia*	*rollottii*	3330	475
Cunoniaceae	*Weinmannia*	*rollottii*	3330	476
Cunoniaceae	*Weinmannia*	*rollottii*	3330	477
Cunoniaceae	*Weinmannia*	*rollottii*	3330	482
Cunoniaceae	*Weinmannia*	*rollottii*	3290	403
Cunoniaceae	*Weinmannia*	*rollottii*	3290	406
Cunoniaceae	*Weinmannia*	*rollottii*	3290	407
Cunoniaceae	*Weinmannia*	*rollottii*	3290	410
Cunoniaceae	*Weinmannia*	*rollottii*	3290	412
Cunoniaceae	*Weinmannia*	*rollottii*	3290	413
Cunoniaceae	*Weinmannia*	*rollottii*	3290	419
Cunoniaceae	*Weinmannia*	*rollottii*	3290	427
Cunoniaceae	*Weinmannia*	*rollottii*	3290	429
Cunoniaceae	*Weinmannia*	*rollottii*	3290	430
Cunoniaceae	*Weinmannia*	*rollottii*	3290	435
Cunoniaceae	*Weinmannia*	*rollottii*	3290	440
Cunoniaceae	*Weinmannia*	*rollottii*	3290	441
Cunoniaceae	*Weinmannia*	*rollottii*	3290	442
Cunoniaceae	*Weinmannia*	*rollottii*	3290	443
Cunoniaceae	*Weinmannia*	*rollottii*	3290	446
Cunoniaceae	*Weinmannia*	*rollottii*	2950	269
Cunoniaceae	*Weinmannia*	*rollottii*	2950	270
Cyatheaceae	*Cyathea*	cf *frigida*	3320	558
Cyatheaceae	*Cyathea*	cf *frigida*	3330	466
Cyatheaceae	*Cyathea*	cf *frigida*	3290	387
Cyatheaceae	*Cyathea*	cf *frigida*	3290	389
Cyatheaceae	*Cyathea*	cf *frigida*	3290	392
Cyatheaceae	*Cyathea*	cf *frigida*	3290	393
Cyatheaceae	*Cyathea*	cf *frigida*	3290	397
Cyatheaceae	*Cyathea*	cf *frigida*	3290	398
Cyatheaceae	*Cyathea*	cf *frigida*	3290	405
Cyatheaceae	*Cyathea*	cf *frigida*	3290	409

Cyatheaceae	*Cyathea*	cf *frigida*	3290	426
Cyatheaceae	*Cyathea*	cf *frigida*	3290	436
Cyatheaceae	*Cyathea*	cf *frigida*	3290	444
Cyatheaceae	*Cyathea*	cf *frigida*	3250	366
Cyatheaceae	*Cyathea*	cf *frigida*	3250	369
Cyatheaceae	*Cyathea*	cf *frigida*	3250	373
Cyatheaceae	*Cyathea*	cf *frigida*	3250	377
Cyatheaceae	*Cyathea*	cf *frigida*	3250	378
Cyatheaceae	*Cyathea*	cf *frigida*	3160	351
Cyatheaceae	*Cyathea*	cf *frigida*	3160	353
Cyatheaceae	*Cyathea*	cf *frigida*	3160	358
Cyatheaceae	*Cyathea*	cf *frigida*	3090	325
Cyatheaceae	*Cyathea*	cf *frigida*	3090	328
Cyatheaceae	*Cyathea*	cf *frigida*	3090	334
Cyatheaceae	*Cyathea*	cf *frigida*	3090	338
Cyatheaceae	*Cyathea*	cf *frigida*	3090	343
Cyatheaceae	*Cyathea*	cf *frigida*	3020	295
Cyatheaceae	*Cyathea*	cf *frigida*	3020	297
Cyatheaceae	*Cyathea*	cf *frigida*	3020	298
Cyatheaceae	*Cyathea*	cf *frigida*	3020	300
Cyatheaceae	*Cyathea*	cf *frigida*	3020	308
Cyatheaceae	*Cyathea*	cf *frigida*	3020	314
Cyatheaceae	*Cyathea*	cf *frigida*	3020	320
Cyatheaceae	*Cyathea*	cf *frigida*	2950	275
Cyatheaceae	*Cyathea*	cf *frigida*	2950	280
Cyatheaceae	*Cyathea*	cf *frigida*	2950	286
Cyatheaceae	*Cyathea*	cf *frigida*	2950	287
Cyatheaceae	*Cyathea*	cf *frigida*	2860	258
Cyatheaceae	*Cyathea*	cf *frigida*	2860	261
Cyatheaceae	*Cyathea*	cf *frigida*	2860	262
Cyatheaceae	*Cyathea*	cf *frigida*	2820	199
Cyatheaceae	*Cyathea*	cf *frigida*	2820	201
Cyatheaceae	*Cyathea*	cf *frigida*	2820	212
Cyatheaceae	*Cyathea*	cf *frigida*	2820	220
Cyatheaceae	*Cyathea*	cf *frigida*	2820	234
Cyatheaceae	*Cyathea*	cf *frigida*	2770	164
Cyatheaceae	*Cyathea*	cf *frigida*	2770	184
Cyatheaceae	*Cyathea*	cf *frigida*	2700	149
Cyatheaceae	*Cyathea*	cf *frigida*	2700	150
Cyatheaceae	*Cyathea*	cf *frigida*	2670	98
Cyatheaceae	*Cyathea*	cf *frigida*	2670	100
Cyatheaceae	*Cyathea*	cf *frigida*	2670	103
Cyatheaceae	*Cyathea*	cf *frigida*	2670	110
Cyatheaceae	*Cyathea*	cf *frigida*	2670	112
Cyatheaceae	*Cyathea*	cf *frigida*	2670	117
Cyatheaceae	*Cyathea*	cf *frigida*	2670	122
Cyatheaceae	*Cyathea*	cf *frigida*	2670	128
Cyatheaceae	*Cyathea*	cf *frigida*	2440	22
Cyatheaceae	*Cyathea*	cf *frigida*	2440	47
Cyatheaceae	*Cyathea*	cf *frigida*	2440	52
Dicksoniaceae	*Dicksonia*	*sellowiana*	2950	266
Dicksoniaceae	*Dicksonia*	*sellowiana*	2950	268

Dicksoniaceae	*Dicksonia*	*sellowiana*	2860	252
Dicksoniaceae	*Dicksonia*	*sellowiana*	2860	259
Ericaceae	*Pernettya*	*prostrata*	3330	478
Escalloniaceae	*Escallonia*	*myrtilloides*	3320	514
Escalloniaceae	*Escallonia*	*myrtilloides*	3330	467
Escalloniaceae	*Escallonia*	*myrtilloides*	3330	484
Escalloniaceae	*Escallonia*	*myrtilloides*	3290	432
Escalloniaceae	*Escallonia*	*myrtilloides*	3290	434
Euphorbiaceae	*Hyeronima*	*macrocarpa*	2670	102
Euphorbiaceae	*Hyeronima*	*scabrida*	3020	313
Euphorbiaceae	*Hyeronima*	*scabrida*	2700	153
Euphorbiaceae	*Sapium*	*laurifolium*	2440	1
Euphorbiaceae	*Sapium*	*laurifolium*	2440	6
Euphorbiaceae	*Sapium*	*laurifolium*	2440	10
Euphorbiaceae	*Sapium*	*laurifolium*	2440	12
Euphorbiaceae	*Sapium*	*laurifolium*	2440	13
Euphorbiaceae	*Sapium*	*laurifolium*	2440	15
Euphorbiaceae	*Sapium*	*laurifolium*	2440	34
Euphorbiaceae	*Sapium*	*laurifolium*	2440	36
Euphorbiaceae	*Sapium*	*stylare*	2700	142
Euphorbiaceae	*Sapium*	*stylare*	2670	106
Euphorbiaceae	*Sapium*	*stylare*	2560	83
Fabaceae	*Inga*	cf *insignis*	2670	99
Fabaceae	*Inga*	cf *insignis*	2560	65
Lamiaceae	*Aegiphila*	*bogotensis*	3090	337
Lauraceae	*Beilschmiedia*	*tovarensis*	2440	26
Lauraceae	*Beilschmiedia*	*tovarensis*	2440	41
Lauraceae	*Endlicheria*	*sp*	2700	134
Lauraceae	*Nectandra*	cf *laurel*	2820	214
Lauraceae	*Nectandra*	cf *laurel*	2770	193
Lauraceae	*Nectandra*	cf *laurel*	2560	67
Lauraceae	*Nectandra*	cf *obtusata*	2950	283
Lauraceae	*Nectandra*	cf *obtusata*	2440	16
Lauraceae	*Nectandra*	*sp*	2820	205
Lauraceae	*Nectandra*	*sp*	2820	207
Lauraceae	*Nectandra*	*sp*	2560	90
Lauraceae	*Nectandra*	*sp*	2560	93
Lauraceae	*Nectandra*	*sp*	2560	95
Lauraceae	*Ocotea*	*sericea*	2670	125
Lauraceae	*Ocotea*	*sericea*	2440	27
Melastomataceae	*Axinaea*	cf *sclerophylla*	3020	292
Melastomataceae	*Axinaea*	cf *sclerophylla*	3020	302
Melastomataceae	*Axinaea*	cf *sclerophylla*	2950	267
Melastomataceae	*Axinaea*	*macrophylla*	3320	565
Melastomataceae	*Axinaea*	*macrophylla*	3330	455
Melastomataceae	*Axinaea*	*macrophylla*	3330	480
Melastomataceae	*Axinaea*	*macrophylla*	3330	483
Melastomataceae	*Axinaea*	*macrophylla*	3290	431
Melastomataceae	*Meriania*	*maxima*	3020	315
Melastomataceae	*Meriania*	*tomentosa*	2700	132
Melastomataceae	*Meriania*	*tomentosa*	2700	133
Melastomataceae	*Meriania*	*tomentosa*	2700	136

Melastomataceae	*Miconia*	cf *sodiroi*	3160	344
Melastomataceae	*Miconia*	cf *sodiroi*	3160	345
Melastomataceae	*Miconia*	cf *sodiroi*	3160	350
Melastomataceae	*Miconia*	cf *sodiroi*	3090	323
Melastomataceae	*Miconia*	cf *sodiroi*	3090	332
Melastomataceae	*Miconia*	cf *sodiroi*	3090	333
Melastomataceae	*Miconia*	cf *sodiroi*	3090	336
Melastomataceae	*Miconia*	cf *sodiroi*	3090	341
Melastomataceae	*Miconia*	cf *sodiroi*	3090	342
Melastomataceae	*Miconia*	cf *sodiroi*	3020	319
Melastomataceae	*Miconia*	cf *sodiroi*	3020	321
Melastomataceae	*Miconia*	*corymbiformis*	3330	451
Melastomataceae	*Miconia*	*corymbiformis*	3020	305
Melastomataceae	*Miconia*	*corymbiformis*	3020	306
Melastomataceae	*Miconia*	*corymbiformis*	3020	307
Melastomataceae	*Miconia*	*corymbiformis*	3020	309
Melastomataceae	*Miconia*	*corymbiformis*	3020	310
Melastomataceae	*Miconia*	*corymbiformis*	2950	281
Melastomataceae	*Miconia*	*corymbiformis*	2950	282
Melastomataceae	*Miconia*	*lasiocalyx*	2770	186
Melastomataceae	*Miconia*	*lasiocalyx*	2770	187
Melastomataceae	*Miconia*	*lasiocalyx*	2770	191
Melastomataceae	*Miconia*	*lasiocalyx*	2700	151
Melastomataceae	*Miconia*	*lasiocalyx*	2670	97
Melastomataceae	*Miconia*	*lasiocalyx*	2560	59
Melastomataceae	*Miconia*	*sp*	3320	519
Melastomataceae	*Miconia*	*sp*	3320	523
Melastomataceae	*Miconia*	*theaezans*	3020	290
Melastomataceae	*Miconia*	*theaezans*	3020	291
Melastomataceae	*Miconia*	*theaezans*	3020	317
Melastomataceae	*Miconia*	*theaezans*	2950	271
Melastomataceae	*Miconia*	*theaezans*	2860	251
Melastomataceae	*Miconia*	*theaezans*	2860	254
Melastomataceae	*Miconia*	*theaezans*	2820	213
Melastomataceae	*Miconia*	*theaezans*	2820	215
Melastomataceae	*Miconia*	*theaezans*	2820	217
Melastomataceae	*Miconia*	*theaezans*	2820	219
Melastomataceae	*Miconia*	*theaezans*	2820	237
Melastomataceae	*Topobea*	cf *acuminata*	2950	272
Melastomataceae	*Topobea*	cf *acuminata*	2950	274
Melastomataceae	*Topobea*	cf *acuminata*	2820	197
Melastomataceae	*Topobea*	cf *acuminata*	2820	200
Melastomataceae	*Topobea*	cf *acuminata*	2820	203
Melastomataceae	*Topobea*	cf *acuminata*	2820	204
Melastomataceae	*Topobea*	cf *acuminata*	2770	160
Melastomataceae	*Topobea*	cf *acuminata*	2770	162
Melastomataceae	*Topobea*	cf *acuminata*	2770	165
Melastomataceae	*Topobea*	cf *acuminata*	2770	167
Melastomataceae	*Topobea*	cf *acuminata*	2770	169
Melastomataceae	*Topobea*	cf *acuminata*	2770	170
Melastomataceae	*Topobea*	cf *acuminata*	2770	173
Melastomataceae	*Topobea*	cf *acuminata*	2770	182

Melastomataceae	*Topobea*	cf *acuminata*	2770	192
Melastomataceae	*Topobea*	cf *acuminata*	2700	131
Melastomataceae	*Topobea*	cf *acuminata*	2700	137
Melastomataceae	*Topobea*	cf *acuminata*	2700	147
Melastomataceae	*Topobea*	cf *acuminata*	2700	155
Melastomataceae	*Topobea*	cf *acuminata*	2670	107
Melastomataceae	*Topobea*	cf *acuminata*	2670	113
Melastomataceae	*Topobea*	cf *acuminata*	2560	55
Melastomataceae	*Topobea*	cf *acuminata*	2560	85
Melastomataceae	*Topobea*	cf *acuminata*	2440	20
Meliaceae	*Guarea*	*kunthiana*	2700	138
Meliaceae	*Guarea*	*kunthiana*	2670	118
Meliaceae	*Guarea*	*kunthiana*	2560	73
Meliaceae	*Ruagea*	*membranacea*	2670	114
Meliaceae	*Ruagea*	*pubescens*	3290	399
Meliaceae	*Ruagea*	*pubescens*	3090	327
Meliaceae	*Ruagea*	*pubescens*	3090	335
Meliaceae	*Ruagea*	*pubescens*	2950	265
Meliaceae	*Ruagea*	*pubescens*	2860	248
Moraceae	*Ficus*	*dulciaria*	2770	178
Moraceae	*Ficus*	*dulciaria*	2700	156
Moraceae	*Ficus*	*dulciaria*	2700	157
Moraceae	*Ficus*	*dulciaria*	2440	11
Moraceae	*Ficus*	*dulciaria*	2440	19
Moraceae	*Ficus*	*dulciaria*	2440	28
Moraceae	*Ficus*	*dulciaria*	2440	51
Myrtaceae	*Myrcianthes*	*orthostemon*	2860	246
Myrtaceae	*Myrcianthes*	*orthostemon*	2860	257
Myrtaceae	*Myrcianthes*	*orthostemon*	2820	222
Myrtaceae	*Myrcianthes*	*orthostemon*	2770	196
Myrtaceae	*Myrcianthes*	*rhopaloides*	2820	227
Myrtaceae	*Myrcianthes*	*rhopaloides*	2560	57
Myrtaceae	*Myrcianthes*	*rhopaloides*	2560	60
Myrtaceae	*Myrcianthes*	*rhopaloides*	2560	69
Myrtaceae	*Myrcianthes*	*rhopaloides*	2560	75
Myrtaceae	*Myrcianthes*	*rhopaloides*	2560	77
Myrtaceae	*Myrcianthes*	*rhopaloides*	2560	80
Myrtaceae	*Myrcianthes*	*rhopaloides*	2560	81
Myrtaceae	*Myrcianthes*	*rhopaloides*	2560	84
Myrtaceae	*Myrcianthes*	*rhopaloides*	2560	89
Myrtaceae	*Myrcianthes*	*rhopaloides*	2440	8
Myrtaceae	*Myrcianthes*	*rhopaloides*	2440	17
Myrtaceae	*Myrcianthes*	*rhopaloides*	2440	23
Myrtaceae	*Myrcianthes*	*rhopaloides*	2440	29
Myrtaceae	*Myrcianthes*	*rhopaloides*	2440	32
Myrtaceae	*Myrcianthes*	*rhopaloides*	2440	33
Myrtaceae	*Myrcianthes*	*rhopaloides*	2440	35
Myrtaceae	*Myrcianthes*	*rhopaloides*	2440	38
Myrtaceae	*Myrcianthes*	*rhopaloides*	2440	39
Myrtaceae	*Myrcianthes*	*rhopaloides*	2440	42
Myrtaceae	*Myrcianthes*	*rhopaloides*	2440	43
Myrtaceae	*Myrcianthes*	*rhopaloides*	2440	48

Myrtaceae	*Myrcianthes*	*rhopaloides*	2440	49
Pentaphylacaceae	*Freziera*	*reticulata*	3290	437
Pentaphylacaceae	*Freziera*	*verrucosa*	3320	510
Pentaphylacaceae	*Freziera*	*verrucosa*	3320	511
Pentaphylacaceae	*Freziera*	*verrucosa*	3320	515
Pentaphylacaceae	*Freziera*	*verrucosa*	3320	520
Pentaphylacaceae	*Freziera*	*verrucosa*	3320	526
Pentaphylacaceae	*Freziera*	*verrucosa*	3320	532
Pentaphylacaceae	*Freziera*	*verrucosa*	3320	539
Pentaphylacaceae	*Freziera*	*verrucosa*	3320	557
Pentaphylacaceae	*Freziera*	*verrucosa*	3320	570
Pentaphylacaceae	*Freziera*	*verrucosa*	3320	575
Pentaphylacaceae	*Freziera*	*verrucosa*	3320	579
Pentaphylacaceae	*Freziera*	*verrucosa*	3320	583
Pentaphylacaceae	*Freziera*	*verrucosa*	3320	593
Pentaphylacaceae	*Freziera*	*verrucosa*	3320	597
Pentaphylacaceae	*Freziera*	*verrucosa*	3330	452
Pentaphylacaceae	*Freziera*	*verrucosa*	3330	456
Pentaphylacaceae	*Freziera*	*verrucosa*	3330	458
Pentaphylacaceae	*Freziera*	*verrucosa*	3330	460
Pentaphylacaceae	*Freziera*	*verrucosa*	3330	465
Pentaphylacaceae	*Freziera*	*verrucosa*	3330	479
Pentaphylacaceae	*Freziera*	*verrucosa*	3330	481
Pentaphylacaceae	*Freziera*	*verrucosa*	3330	496
Pentaphylacaceae	*Freziera*	*verrucosa*	3290	411
Pentaphylacaceae	*Freziera*	*verrucosa*	3290	414
Pentaphylacaceae	*Freziera*	*verrucosa*	3290	415
Pentaphylacaceae	*Freziera*	*verrucosa*	3290	416
Pentaphylacaceae	*Freziera*	*verrucosa*	3290	418
Pentaphylacaceae	*Freziera*	*verrucosa*	3290	445
Pentaphylacaceae	*Ternstroemia*	*lehmannii*	2860	249
Pentaphylacaceae	*Ternstroemia*	*lehmannii*	2820	230
Pentaphylacaceae	*Ternstroemia*	*lehmannii*	2770	185
Pentaphylacaceae	*Ternstroemia*	*lehmannii*	2670	111
Pentaphylacaceae	*Ternstroemia*	*lehmannii*	2670	126
Pentaphylacaceae	*Ternstroemia*	*lehmannii*	2560	76
Piperaceae	*Piper*	*puraceanum*	3020	312
Piperaceae	*Piper*	*sodiroi*	2700	129
Piperaceae	*Piper*	*sodiroi*	2560	78
Primulaceae	*Ardisia*	*foetida*	2820	211
Primulaceae	*Ardisia*	*foetida*	2820	223
Primulaceae	*Cybianthus*	*sp*	3290	438
Primulaceae	*Geissanthus*	*andinus*	3320	498
Primulaceae	*Geissanthus*	*andinus*	3320	508
Primulaceae	*Geissanthus*	*andinus*	3320	521
Primulaceae	*Geissanthus*	*andinus*	3320	535
Primulaceae	*Geissanthus*	*andinus*	3320	545
Primulaceae	*Geissanthus*	*andinus*	3320	550
Primulaceae	*Geissanthus*	*andinus*	3320	564
Primulaceae	*Geissanthus*	*andinus*	3320	566
Primulaceae	*Geissanthus*	*andinus*	3320	571
Primulaceae	*Geissanthus*	*andinus*	3320	584

Primulaceae	*Geissanthus*	*andinus*	3290	385
Primulaceae	*Geissanthus*	*andinus*	3290	386
Primulaceae	*Geissanthus*	*andinus*	3290	395
Primulaceae	*Geissanthus*	*andinus*	3290	417
Primulaceae	*Geissanthus*	*andinus*	3290	421
Primulaceae	*Geissanthus*	*ecuadorensis*	3320	560
Primulaceae	*Geissanthus*	*ecuadorensis*	3160	348
Primulaceae	*Geissanthus*	*ecuadorensis*	2770	176
Primulaceae	*Geissanthus*	*ecuadorensis*	2700	135
Primulaceae	*Geissanthus*	*ecuadorensis*	2700	144
Primulaceae	*Geissanthus*	*vanderwerffii*	3330	448
Primulaceae	*Geissanthus*	*vanderwerffii*	3330	450
Primulaceae	*Geissanthus*	*vanderwerffii*	3290	423
Primulaceae	*Myrsine*	*coriacea*	2440	18
Rosaceae	*Hesperomeles*	*obtusifolia*	3320	506
Rosaceae	*Hesperomeles*	*obtusifolia*	3320	507
Rosaceae	*Prunus*	*huantensis*	3290	384
Rosaceae	*Prunus*	*huantensis*	3290	394
Rosaceae	*Prunus*	*huantensis*	3290	422
Rosaceae	*Prunus*	*huantensis*	3250	371
Rosaceae	*Prunus*	*huantensis*	3250	372
Rosaceae	*Prunus*	*huantensis*	3160	349
Rosaceae	*Prunus*	*huantensis*	3160	360
Rosaceae	*Prunus*	*huantensis*	3020	293
Rubiaceae	*Cinchona*	*pitayensis*	3160	354
Rubiaceae	*Cinchona*	*pitayensis*	3160	363
Rubiaceae	*Cinchona*	*pitayensis*	2670	127
Rubiaceae	*Faramea*	*calyptrata*	2560	54
Rubiaceae	*Faramea*	*calyptrata*	2560	56
Rubiaceae	*Faramea*	*calyptrata*	2560	62
Rubiaceae	*Faramea*	*calyptrata*	2560	63
Rubiaceae	*Faramea*	*calyptrata*	2560	64
Rubiaceae	*Faramea*	*calyptrata*	2560	68
Rubiaceae	*Faramea*	*calyptrata*	2560	72
Rubiaceae	*Faramea*	*calyptrata*	2560	74
Rubiaceae	*Faramea*	*calyptrata*	2560	82
Rubiaceae	*Faramea*	*calyptrata*	2560	86
Rubiaceae	*Faramea*	*calyptrata*	2560	87
Rubiaceae	*Faramea*	*calyptrata*	2560	88
Rubiaceae	*Faramea*	*calyptrata*	2560	92
Rubiaceae	*Faramea*	*calyptrata*	2560	94
Rubiaceae	*Faramea*	*calyptrata*	2440	7
Rubiaceae	*Faramea*	*calyptrata*	2440	14
Rubiaceae	*Faramea*	*calyptrata*	2440	37
Rubiaceae	*Faramea*	*calyptrata*	2440	45
Rubiaceae	*Faramea*	*calyptrata*	2440	53
Rubiaceae	*Faramea*	cf *ovalis*	2820	209
Rubiaceae	*Faramea*	cf *ovalis*	2770	171
Rubiaceae	*Faramea*	cf *ovalis*	2770	174
Rubiaceae	*Faramea*	cf *ovalis*	2770	179
Rubiaceae	*Faramea*	cf *ovalis*	2770	181
Rubiaceae	*Faramea*	cf *ovalis*	2770	188

Rubiaceae	*Faramea*	cf *ovalis*	2770	189
Rubiaceae	*Faramea*	cf *ovalis*	2770	195
Rubiaceae	*Faramea*	*flavicans*	2950	277
Rubiaceae	*Faramea*	*flavicans*	2860	244
Rubiaceae	*Faramea*	*flavicans*	2820	232
Rubiaceae	*Faramea*	*flavicans*	2820	239
Rubiaceae	*Faramea*	*flavicans*	2770	166
Rubiaceae	*Faramea*	*flavicans*	2770	168
Rubiaceae	*Guettarda*	*crispiflora*	2440	50
Rubiaceae	*Guettarda*	*hirsuta*	2700	141
Rubiaceae	*Palicourea*	*amethystina*	3250	380
Rubiaceae	*Palicourea*	*amethystina*	3250	382
Rubiaceae	*Palicourea*	*amethystina*	3160	365
Rubiaceae	*Palicourea*	*amethystina*	3020	301
Rubiaceae	*Palicourea*	*amethystina*	2700	143
Rubiaceae	*Palicourea*	*amethystina*	2670	101
Rubiaceae	*Palicourea*	*amethystina*	2670	105
Rubiaceae	*Palicourea*	*amethystina*	2670	115
Rubiaceae	*Palicourea*	*amethystina*	2670	116
Rubiaceae	*Palicourea*	*amethystina*	2670	121
Rubiaceae	*Palicourea*	*amethystina*	2670	124
Rubiaceae	*Palicourea*	cf *stipularis*	3020	296
Rubiaceae	*Palicourea*	cf *stipularis*	2560	61
Rubiaceae	*Palicourea*	cf *stipularis*	2560	91
Rubiaceae	*Palicourea*	*stenosepala*	2440	9
Rubiaceae	*Palicourea*	*stenosepala*	2440	25
Rutaceae	*Zanthoxylum*	*andinum*	2950	278
Sabiaceae	*Meliosma*	*arenosa*	3020	304
Sabiaceae	*Meliosma*	*arenosa*	2820	231
Sabiaceae	*Meliosma*	*arenosa*	2820	233
Sabiaceae	*Meliosma*	*arenosa*	2700	146
Sabiaceae	*Meliosma*	*frondosa*	3160	355
Sabiaceae	*Meliosma*	*frondosa*	3160	364
Sabiaceae	*Meliosma*	*frondosa*	2860	263
Sabiaceae	*Meliosma*	*frondosa*	2820	238
Salicaceae	*Casearia*	*sylvestris*	2440	3
Salicaceae	*Casearia*	*sylvestris*	2440	4
Salicaceae	*Casearia*	*sylvestris*	2440	31
Sapindaceae	*Allophylus*	*excelsus*	2860	243
Siparunaceae	*Siparuna*	*piloso-lepidota*	3160	346
Siparunaceae	*Siparuna*	*piloso-lepidota*	3090	329
Solanaceae	*Cestrum*	*humboldtii*	3250	368
Solanaceae	*Solanum*	*sp2*	3090	340
Solanaceae	*Solanum*	*sp2*	2440	46
Solanaceae	*Solanum*	*sp2*	2820	218
Solanaceae	*Solanum*	*sp3*	3090	339
Styracaceae	*Styrax*	*heterotrichus*	2820	206
Styracaceae	*Styrax*	*heterotrichus*	2820	208
Styracaceae	*Styrax*	*heterotrichus*	2820	225
Symplocaceae	*Symplocos*	*quitensis*	2770	158
Symplocaceae	*Symplocos*	*quitensis*	2670	108
Symplocaceae	*Symplocos*	*subandina*	3250	379

Symplocaceae	*Symplocos*	*subandina*	3250	381
Theaceae	*Gordonia*	*fruticosa*	3330	497
Theaceae	*Gordonia*	*fruticosa*	3290	383
Theaceae	*Gordonia*	*fruticosa*	3290	388
Theaceae	*Gordonia*	*fruticosa*	3290	391
Theaceae	*Gordonia*	*fruticosa*	3290	396
Theaceae	*Gordonia*	*fruticosa*	3290	400
Theaceae	*Gordonia*	*fruticosa*	3290	401
Theaceae	*Gordonia*	*fruticosa*	3290	404
Theaceae	*Gordonia*	*fruticosa*	3290	408
Theaceae	*Gordonia*	*fruticosa*	3290	424
Theaceae	*Gordonia*	*fruticosa*	3290	425
Theaceae	*Gordonia*	*fruticosa*	3090	324
Theaceae	*Gordonia*	*fruticosa*	3020	316
Theaceae	*Gordonia*	*fruticosa*	3020	318
Theaceae	*Gordonia*	*fruticosa*	2950	276
Theaceae	*Gordonia*	*fruticosa*	2860	241
Theaceae	*Gordonia*	*fruticosa*	2860	247
Theaceae	*Gordonia*	*fruticosa*	2860	250
Theaceae	*Gordonia*	*fruticosa*	2860	256
Theaceae	*Gordonia*	*fruticosa*	2860	260
Theaceae	*Gordonia*	*fruticosa*	2820	224
Theaceae	*Gordonia*	*fruticosa*	2820	229
Theaceae	*Gordonia*	*fruticosa*	2820	236
Theaceae	*Gordonia*	*fruticosa*	2770	175
Theaceae	*Gordonia*	*fruticosa*	2770	180
Theaceae	*Gordonia*	*fruticosa*	2770	183
Theaceae	*Gordonia*	*fruticosa*	2770	190
Theaceae	*Gordonia*	*fruticosa*	2700	130
Theaceae	*Gordonia*	*fruticosa*	2670	104
Urticaceae	*Cecropia*	*gabrielis*	2440	44

Table S2. Specimen information for samples included in the phylogenetic analyses. A sample ID is given if the sequence was obtained from a specimen collected in this study. All specimens are deposited at the Herbario-QCA at Pontificia Universidad Católica del Ecuador. Sequences can be found on the Barcode of Life Data System (BOLD) and GenBank.

Family	Genus	Specific Epithet	Sample ID	*rbc*L	*mat*K
Adoxaceae	*Viburnum*	*urbanii*	RJP-T10-226	Yes	Yes
Aquifoliaceae	*Ilex*	*hualgayoca*	RJP-T1-553	Yes	Yes
Aquifoliaceae	*Ilex*	*myricoides*	RJP-T2-474	Yes	Yes
Aquifoliaceae	*Ilex*	*weberlingii*	RJP-T1-587	Yes	Yes
Araliaceae	*Oreopanax*	*grandifolius*	RJP-T9-253	Yes	Yes
Araliaceae	*Oreopanax*	*palamophyllus*	RJP-T12-140	Yes	Yes
Asteraceae	*Asteraceae*	*sp*	RJP-T10-202	Yes	Yes
Brunelliaceae	*Brunellia*	*tomentosa*	RLJ-11412	Yes	Yes
Chloranthaceae	*Hedyosmum*	*cuatrecazanum*	RJP-T11-194	Yes	No
Clethraceae	*Clethra*	*ovalifolia*	RLJ-11176	Yes	Yes
Clusiaceae	*Clusia*	*sp2*	RJP-T14-70	Yes	No
Clusiaceae	*Clusia*	*sp3*	RJP-T9-255	Yes	No
Cunoniaceae	*Weinmannia*	*auriculifera*	RJP-T8-284	Yes	Yes
Cunoniaceae	*Weinmannia*	*lentiscifolia*	RJP-T10-198	Yes	Yes

Cunoniaceae	*Weinmannia*	*mariquitae*	RJP-T1-573	Yes	No
Cunoniaceae	*Weinmannia*	*pinnata*	RLJ-11031	Yes	Yes
Cunoniaceae	*Weinmannia*	*rollottii*	RJP-T8-269	Yes	Yes
Cyatheaceae	*Cyathea*	cf *frigida*	RJP-T13-100	Yes	No
Dicksoniaceae	*Dicksonia*	*sellowiana*	RJP-T9-252	Yes	No
Ericaceae	*Pernettya*	*prostrata*	RJP-T2-478	Yes	No
Escalloniaceae	*Escallonia*	*myrtilloides*	RJP-T1-514	Yes	Yes
Euphorbiaceae	*Hyeronima*	*macrocarpa*	RLJ-11149	Yes	No
Euphorbiaceae	*Sapium*	*stylare*	RJP-T14-83	Yes	Yes
Fabaceae	*Inga*	cf *insignis*	RJP-T13-99	Yes	Yes
Lamiaceae	*Aegiphila*	*bogotensis*	RJP-T6-337	Yes	Yes
Lauraceae	*Beilschmiedia*	*tovarensis*	RLJ-11338	Yes	Yes
Lauraceae	*Endlicheria*	*sp*	RJP-T12-134	Yes	No
Lauraceae	*Nectandra*	cf *obtusata*	RJP-T15-16	Yes	Yes
Lauraceae	*Nectandra*	*sp*	RJP-T14-93	Yes	Yes
Lauraceae	*Ocotea*	*sericea*	RJP-T15-27	Yes	Yes
Melastomataceae	*Meriania*	*maxima*	RJP-T7-315	Yes	No
Melastomataceae	*Meriania*	*tomentosa*	RJP-T12-132	Yes	No
Melastomataceae	*Miconia*	cf *sodiroi*	RJP-T7-319	Yes	No
Melastomataceae	*Miconia*	*corymbiformis*	RLJ-11260	Yes	No
Melastomataceae	*Miconia*	*lasiocalyx*	RJP-T14-59	Yes	No
Melastomataceae	*Topobea*	cf *acuminata*	RJP-T14-55	Yes	No
Meliaceae	*Guarea*	*kunthiana*	RJP-T14-73	Yes	Yes
Meliaceae	*Ruagea*	*membranacea*	RJP-T13-114	Yes	Yes
Meliaceae	*Ruagea*	*pubescens*	RJP-T3-399	Yes	Yes
Moraceae	*Ficus*	*dulciaria*	RJP-T15-19	Yes	Yes
Myrtaceae	*Myrcianthes*	*orthostemon*	RJP-T10-222	Yes	Yes
Myrtaceae	*Myrcianthes*	*rhopaloides*	RJP-T14-57	Yes	No
Pentaphylacaceae	*Freziera*	*verrucosa*	RLJ-11262	Yes	Yes
Pentaphylacaceae	*Ternstroemia*	*lehmannii*	RJP-T11-185	Yes	Yes
Piperaceae	*Piper*	*puraceanum*	RJP-T7-312	Yes	Yes
Piperaceae	*Piper*	*sodiroi*	RJP-T12-129	Yes	No
Primulaceae	*Ardisia*	*foetida*	RJP-T10-211	Yes	Yes
Primulaceae	*Geissanthus*	*andinus*	RJP-T1-498	Yes	Yes
Primulaceae	*Geissanthus*	*ecuadorensis*	RJP-T5-348	Yes	Yes
Primulaceae	*Myrsine*	*coriacea*	RLJ-11059	Yes	Yes
Rosaceae	*Hesperomeles*	*obtusifolia*	RLJ-11247	Yes	Yes
Rosaceae	*Prunus*	*huantensis*	RJP-T7-293	Yes	Yes
Rubiaceae	*Cinchona*	*pitayensis*	RJP-T13-127	Yes	Yes
Rubiaceae	*Faramea*	*calyptrata*	RJP-T14-54	Yes	Yes
Rubiaceae	*Faramea*	cf *ovalis*	RJP-T11-171	Yes	Yes
Rubiaceae	*Faramea*	*flavicans*	RJP-T10-239	Yes	Yes
Rubiaceae	*Guettarda*	*crispiflora*	NA	Yes	Yes
Rubiaceae	*Palicourea*	*amethystina*	RLJ-11124	Yes	Yes
Rubiaceae	*Palicourea*	cf *stipularis*	RJP-T14-61	Yes	Yes
Rutaceae	*Zanthoxylum*	*andinum*	RJP-T8-278	Yes	No
Sabiaceae	*Meliosma*	*arenosa*	RJP-T12-146	Yes	Yes
Sabiaceae	*Meliosma*	*frondosa*	RJP-T9-263	Yes	Yes
Salicaceae	*Casearia*	*sylvestris*	RJP-T15-31	Yes	Yes
Sapindaceae	*Allophylus*	*excelsus*	RJP-T9-243	Yes	No
Siparunaceae	*Siparuna*	*piloso-lepidota*	RJP-T6-329	Yes	Yes
Solanaceae	*Cestrum*	*humboldtii*	RJP-T4-368	Yes	Yes

Solanaceae	*Solanum*	*sp2*	RJP-T10-218	Yes	Yes
Styracaceae	*Styrax*	*heterotrichus*	RJP-T10-206	Yes	Yes
Theaceae	*Gordonia*	*fruticosa*	RJP-T7-316	Yes	Yes
Urticaceae	*Cecropia*	*gabrielis*	RLJ-11223	Yes	Yes

Table S3. Standard effects sizes for phylogenetic diversity (PD) randomizations for each plot. All randomizations were run 999 times. Significant values are denoted by *.

Plot	ntaxa	pd.obs	rand.mean	rand.sd	obs.rank	obs.z	obs.p
1	14	1.438	1.195	0.243	752	0.997	0.752
2	12	1.241	1.062	0.237	695	0.757	0.695
3	15	1.555	1.255	0.246	835	1.218	0.835
4	15	1.667	1.245	0.249	956	1.695	0.956 *
5	12	1.283	1.060	0.233	757	0.960	0.757
6	20	1.483	1.541	0.258	465	−0.224	0.465
7	12	1.292	1.059	0.233	751	1.001	0.751
8	12	1.179	1.064	0.232	693	0.497	0.693
9	11	1.290	1.006	0.233	804	1.218	0.804
10	9	1.270	0.852	0.217	950	1.930	0.950
11	11	1.487	0.993	0.226	987	2.189	0.987 *
12	7	1.054	0.708	0.200	892	1.724	0.892
13	11	1.159	0.998	0.228	719	0.704	0.719
14	14	1.095	1.182	0.247	496	−0.354	0.496
15	9	1.025	0.852	0.218	770	0.797	0.770

Table S4. Standard effects sizes for mean pairwise distance (MPD) randomizations for each plot. All randomizations were run 999 times. Significant values are denoted by *..

Plot	ntaxa	mpd.obs	rand.mean	rand.sd	obs.rank	obs.z	obs.p
1	14	0.246	0.193	0.047	887	1.107	0.887
2	12	0.186	0.183	0.052	663	0.051	0.663
3	15	0.358	0.197	0.045	988	3.577	0.988 *
4	15	0.253	0.207	0.039	875	1.176	0.875
5	12	0.210	0.198	0.048	756	0.250	0.756
6	20	0.260	0.212	0.035	897	1.385	0.897
7	12	0.321	0.198	0.045	966	2.717	0.966 *
8	12	0.344	0.203	0.042	995	3.365	0.995 *
9	11	0.350	0.193	0.048	988	3.252	0.988 *
10	9	0.349	0.186	0.052	971	3.148	0.971 *
11	11	0.313	0.205	0.044	983	2.442	0.983 *
12	7	0.365	0.183	0.052	988	3.481	0.988 *
13	11	0.273	0.192	0.049	901	1.635	0.901
14	14	0.124	0.182	0.048	20	−1.196	0.020 *
15	9	0.128	0.166	0.054	167	−0.697	0.167

Table S5. Standard effects sizes for mean nearest taxon distance (MNTD) randomizations for each plot. All randomizations were run 999 times. Significant values are denoted by *.

Plot	ntaxa	mntd.obs	rand.mean	rand.sd	obs.rank	obs.z	obs.p
1	14	0.160	0.106	0.038	941	1.425	0.941
2	12	0.106	0.112	0.043	463	−0.139	0.463
3	15	0.221	0.103	0.032	991	3.652	0.991 *
4	15	0.127	0.102	0.029	833	0.863	0.833
5	12	0.079	0.112	0.034	147	−0.956	0.147

6	20	0.116	0.088	0.022	889	1.250	0.889
7	12	0.079	0.114	0.034	122	−1.007	0.122
8	12	0.050	0.113	0.031	14	−2.023	0.014 *
9	11	0.205	0.117	0.038	962	2.304	0.962 *
10	9	0.281	0.127	0.040	995	3.883	0.995 *
11	11	0.218	0.118	0.033	995	3.034	0.995 *
12	7	0.282	0.140	0.044	992	3.188	0.992 *
13	11	0.153	0.118	0.036	862	0.981	0.862
14	14	0.025	0.106	0.041	1	−2.004	0.001 *
15	9	0.098	0.127	0.049	243	−0.600	0.243

Table S6. Standard effects sizes for phylogenetic diversity (PD) randomizations for each plot excluding ferns from the data. All randomizations were run 999 times. Significant values are denoted by *.

Plot	ntaxa	pd.obs	rand.mean	rand.sd	obs.rank	obs.z	obs.p
1	13	0.94	0.983	0.104	340	−0.419	0.340
2	12	1.039	0.926	0.101	866	1.117	0.866
3	14	1.066	1.032	0.109	608	0.314	0.608
4	14	1.175	1.037	0.105	915	1.318	0.915
5	11	0.791	0.873	0.100	197	−0.824	0.197
6	19	0.99	1.272	0.111	9	−2.539	0.009 *
7	10	0.78	0.817	0.101	369	−0.372	0.369
8	10	0.669	0.815	0.101	74	−1.448	0.074
9	10	0.795	0.816	0.102	415	−0.211	0.415
10	8	0.777	0.693	0.094	818	0.890	0.818
11	10	0.994	0.810	0.101	965	1.830	0.965 *
12	6	0.556	0.546	0.088	543	0.109	0.543
13	10	0.662	0.811	0.102	69	−1.466	0.069
14	13	0.597	0.984	0.108	2	−3.575	0.002 *
15	8	0.528	0.688	0.097	41	−1.658	0.041 *

Table S7. Standard effects sizes for mean pairwise distance (MPD) randomizations for each plot excluding ferns from the data. All randomizations were run 999 times. Significant values are denoted by *.

Plot	ntaxa	mpd.obs	rand.mean	rand.sd	obs.rank	obs.z	obs.p
1	13	0.180	0.168	0.020	742	0.619	0.742
2	12	0.187	0.162	0.022	875	1.157	0.875
3	14	0.190	0.180	0.018	701	0.526	0.701
4	14	0.190	0.184	0.018	640	0.329	0.640
5	11	0.162	0.174	0.021	294	−0.558	0.294
6	19	0.147	0.192	0.015	3	−3.074	0.003 *
7	10	0.143	0.172	0.021	75	−1.415	0.075
8	10	0.152	0.182	0.019	65	−1.523	0.065
9	10	0.177	0.172	0.021	605	0.249	0.605
10	8	0.176	0.161	0.022	764	0.705	0.764
11	10	0.207	0.181	0.019	913	1.357	0.913
12	6	0.160	0.164	0.024	438	−0.200	0.438
13	10	0.122	0.168	0.022	21	−2.030	0.021 *
14	13	0.115	0.164	0.021	12	−2.326	0.012 *
15	8	0.107	0.148	0.026	58	−1.543	0.058

Table S8. Standard effects sizes for mean nearest taxon distance (MNTD) randomizations for each plot excluding ferns from the data. All randomizations were run 999 times. Significant values are denoted by *.

Plot	ntaxa	mntd.obs	rand.mean	rand.sd	obs.rank	obs.z	obs.p
1	13	0.127	0.099	0.027	847	1.034	0.847
2	12	0.106	0.102	0.030	532	0.126	0.532
3	14	0.098	0.095	0.023	533	0.125	0.533
4	14	0.091	0.094	0.021	431	−0.144	0.431
5	11	0.052	0.106	0.027	23	−2.007	0.023 *
6	19	0.042	0.080	0.016	8	−2.390	0.008 *
7	10	0.099	0.112	0.028	300	−0.500	0.300
8	10	0.061	0.111	0.025	24	−2.019	0.024 *
9	10	0.081	0.113	0.029	145	−1.096	0.145
10	8	0.172	0.122	0.033	938	1.508	0.938
11	10	0.16	0.111	0.024	984	2.030	0.984 *
12	6	0.113	0.139	0.033	212	−0.762	0.212
13	10	0.051	0.112	0.029	25	−2.083	0.025 *
14	13	0.019	0.100	0.029	1	−2.776	0.001 *
15	8	0.087	0.124	0.037	161	−0.998	0.161

Table S9. Forward and reverse primer sequences for the *rbc*L and *mat*K gene regions used for sequencing in this study. References are included for the original publication of each primer.

Primer Name	Direction	Sequence	Reference
rbcLa_F	Forward	ATGTCACCACAAACAGAGACTAAAGC	Levin et al. 2002
rbcLa_R	Reverse	GTAAAATCAAGTCCACCRCG	Kress et al. 2009
matK-xf	Forward	TAATTTACGATCAATTCATTC	Ford et al. 2009
matK-MALP	Reverse	ACAAGAAAGTCGAAGTAT	Dunning and Savolainen 2010

© 2019 by the authors. Licensee MDPI, Basel, Switzerland. This article is an open access article distributed under the terms and conditions of the Creative Commons Attribution (CC BY) license (http://creativecommons.org/licenses/by/4.0/).
